# New Spectrophotometric Method for Simultaneous determination of Metoprolol Tartarate and Hydrochlorthiazide in Tablets

**DOI:** 10.4103/0250-474X.44606

**Published:** 2008

**Authors:** K. R. Gupta, M. R. Tajne, S. G. Wadodkar

**Affiliations:** Department of Pharmaceutical Sciences, RTM Nagpur University, Nagpur-33, India; 1 S. K. B. College of Pharmacy, New Kamptee-441 002, India

**Keywords:** Hydrochlothiazide, metoprolol tartrate, spectrophotometry, two wavelength method

## Abstract

The present work describes a two-wavelength method for simultaneous determination of metoprolol and hydrochlorthiazide in fixed dose combination tablet. The wavelengths selected for method were 257.8 nm, 282.9 nm and 315.0 nm. The absorbance difference at first two wavelengths was used for determination of metoprolol and the latter was used for determination of hydrochlorthiazide. The recovery value for the drugs from the tablet matrix was found to be 100.55% (metoprolol) and 99.97% (comparison with standard) and 98.09% (E1%, 1cm) for hydrochlorthiazide. The method has an advantage that hydrochlorthiazide can be estimated in combination, as there is no interference of metoprolol at 315.0 nm. The method was evaluated statistically for its accuracy and precision.

Metoprolol tartrate (MT) and hydrochlorthiazide (HTZ) are widely used in the treatment of hypertension, cardiac and renal diseases. Tablet formulation containing metoprolol tartarate (MT, 100 mg) and hydrochlorthiazide (HTZ, 12.5 mg) are available in the market and indicated for hypertension. Metoprolol tartrate is, 1-[4-(2-methoxy ethyl) phenoxy]-3-[(1-methyl ethyl) amino]-2-propanol and official in IP^1^ while hydrochlorthiazide is chemically, 6-chloro-3,4-dihydro-2H-1,2,4-benzothiadiazine-7-sulfonamide and official in IP^2^ and USP^3^. Various methods have been reported in the literature for their estimation in dosage forms and biological fluids which includes spectrophotometry^4^, HPLC^5^ and GC-MS^6^ for metoprolol and spectrophotometry^7^,^8^ and HPLC^9^–^11^ for hydrochlorthiazide. A spectrophotomteric^12^ method has been reported in the literature for its simultaneous determination in tablets. The present work describes a new simultaneous spectrophotometric method for their estimation based on two wavelengths absorbance correction in overlapping spectral region for estimation of metoprolol and absorbance measurement at non-overlapping region for estimation of hydrochlorthiazide. The present work has got an advantage over the previous method that hydrochlorthiazide can be estimated in combination with ease, as there is no interference of metoprolol at 315.0 nm.

A Shimadzu double beam spectrophotometer model 1601 with optional package programme and matched pair of quartz 1.0 cm cell. All the chemicals used during the experimentation were of AR grade. A stock solution of MT was prepared in methanol to get a concentration of 1 mg/ml. The stock solution was further appropriately diluted with distilled water to get a concentration of 40 μg/ml solution. Similarly stock solution of HTZ was prepared in methanol to get a concentration of 1 mg/ml. The stock solution was further appropriately diluted with distilled water to get a concentration of 5 μg/ml solution. Both the diluted solutions were scanned in the range of 400-200 nm against reagent blank. The overlain spectrum was recorded and is shown in the fig. 1.

**Fig. 1:**
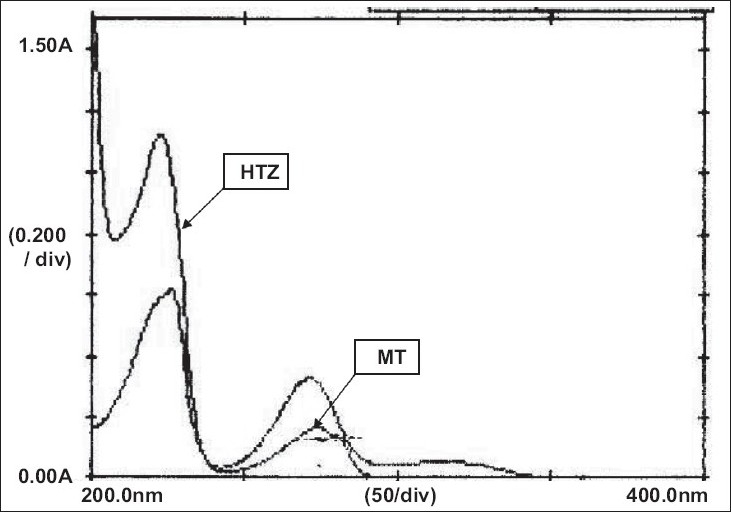
Wavelength selection from overlain spectrum. HTZ indicates hydrochlorthiazide and MT metoprolol tartrate

A solution of hydrochlorthiazide (10 μg/ml) was prepared and E (1%, 1 cm) value was determined at 315 nm (no interference of metoprolol). This value was found to be 105.54. It was calculated using the formula, E (1%, 1 cm)= Absorbance/concentration (g/100 ml).

Stock solutions of MT (1 mg/ml) and HTZ (1 mg/ml) were prepared in methanol. Solutions ranging from 2.5 ml to 12.5 ml from each stock were taken in a series of flask and diluted with distilled water to get a concentration in the range of 0-125 μg/ml for both the drugs and absorbance was measured at 257.8 nm, 282.9 and 315.0 nm against blank. Also the mixture of drugs was found to obey in the range of 40-200 μg/ml for MT and 5-25 μg/ml for HTZ. A linear plot of difference in the absorbance’s (A_1_-A_2_ ) of mixture at 257.8 nm and 282.9 nm was found to obey in the range 40-200 μg/ml.

Both the drugs were weighed in the ratio as in marketed formulation i.e. 8:1, MT (∼100 mg) and (∼12.5 mg) and were transferred to a volumetric flask and the mixture dissolved in methanol and volume made up to mark. A 25 ml portion of this solution was transferred to 50 ml volumetric flask and volume adjusted up to mark with distilled water. A 5 ml portion of this solution was further diluted to 50 ml with distilled water. Similarly five different samples were weighed in the ratio of 8:1 and same procedure was followed as above to get nearly same concentration as in standard solution. The absorbances of the final diluted standard and samples were read at 257.8 0 nm, 282.9 0 nm and 315.0 0 nm

The amounts of drugs were estimated using suitable formulae. For estimation of MT, the formula used was, Cu = [Au/As]×Cs×d --(1), where, Cu = concentration of unknown, Cs= concentration of standard, As= [A_1_-A_2_ ], where, A_1_ and A_2_ are absorbance of standard MT at 257.8 and 282.9 nm, respectively. Au= [A_1_-A_2_ ], where, A_1_ and A_2_ =Absorbance of unknown at 257.8 nm and 282.9 nm and d= dilution factor. Further the percentage was estimated using formula, Percent (estimated)= (amount estimated/actual amount present)×100 --(2). Similarly for estimation of HTZ, (i) By comparison with standard: Cu= [Au/As]×Cs×d --(3), where Cu = concentration of unknown, Cs= concentration of standard, As=absorbance of standard HTZ at 315.0 nm, Au=Absorbance of unknown at 315.0 nm and d= dilution factor. (ii) By E (1%, 1 cm) value, Percent (estimated) = Au×d×100/weight of sample (g) ×E (1%, 1 cm) --(4), where Au=Abs of unknown at 315.0 nm, d= dilution factor and E (1%, 1cm) =105.54.

Twenty tablets were weighed and average weight calculated. Tablets were finely powdered and accurately weighed quantity of powder equivalent to 100 mg of MT was transferred in 50 ml volumetric flask and rest procedure followed as described for laboratory mixture. The estimation of MT in the formulation was done using the formula 1. The estimation of HTZ was done i) By comparison with standard using formula 3 and (ii) by E (1%, 1 cm) was calculated using following formula, concentration (g/100 ml)= Au×d/E (1%, 1 cm) --(5) The results obtained are shown in Table 1.

**TABLE 1:** RESULTS OF ESTIMATION AND RECOVERY STUDIES

Sample code	Statistical parameter	% Labeled claim* % Recovery*					
		
		MT	HTZ		MT	HTZ	
					
		Au/AS	Au/AS	E (1%, 1 cm)	Au/AS	Au/AS	E (1%, 1 cm)
Standard Laboratory Mixture	Mean	99.47	100.09	99.82	-	-	-
	±SD	0.853	1.042	1.018	-	-	-
	CV	0.860	1.040	0.470	-	-	-
	SE	0.380	0.470	0.460	-	-	-
Marketed formulation	Mean	98.92	99.71	98.83	100.55	99.98	98.73
	±SD	0.961	1.440	1.430	0.990	2.80	2.68
	CV	0.970	1.450	1.450	0.990	2.80	2.71
	SE	0.480	0.720	0.720	0.500	1.40	1.34

* Each determination is mean of five observations, MT is metoprolol tartarate and HTZ is hydrochlorthiazide

To study the accuracy of the method, recovery study was performed by standard addition method. The recovery studies were performed at three different levels. Percent recovery was calculated using formula,% recovery= (T-A)/S ×100 --(6), where, T is the total amount of drug estimated, A is the amount contributed by tablet powder (as per amount estimated by proposed method) and S, the amount of pure drug added. The results of recovery studies are shown in Table 1.

From the fig. 1, the wavelengths selected for estimation were 257.8, 282.9 and 315 nm. These wavelengths were selected such that at first two selected wavelengths hydrochlorthiazide shows same absorbance and at latter wavelength metoprolol tartrate shows zero absorbance. The plot of difference in absorbance at 257.8 and 282.9 nm of metoprolol and absorbance of hydrochlorthiazide at 315.0 nm were found to obey Beer-Lambert’s law in the concentration range 0-200 μg/ml with correlation coefficient of 0.9997 and slope 0.0055 and 0-25 μg/ml with correlation coefficient of 0.9999 and slope 0.0107, respectively. The results of estimation were found to be 99.47% for metoprolol and 100.09% (comparison with standard) and 99.82% (E1%,1 cm) for hydrochlorthiazide. The recovery values of the drug indicate the accuracy of the method. The added advantage is that hydrochlorthiazide can be easily estimated by the proposed method without the interference of metoprolol tartrate present in the tablet.
